# Translation, Cross-Cultural Adaptation, and Validation of the Fear of Dependency Scale Into European Portuguese

**DOI:** 10.1177/01939459241273400

**Published:** 2024-08-20

**Authors:** Patrícia Maria Pires, Joana Carvalho, Telma Pires, Carlos Pires, Oscar Ribeiro

**Affiliations:** 1 Department of Health, University of Tras-os-Montes e Alto Douro, Vila Real, Portugal; 2 Clinical Academic Center of Trás-os-Montes e Alto Douro—Doctor Professor Nuno Grande—CACTMAD, Vila Real, Portugal; 3 Faculty of Sports, University of Porto, Porto, Portugal; 4 Research Center in Physical Activity, Health and Leisure (CIAFEL), Faculty of Sport, University of Porto, Porto, Portugal; 5 Hospital Center Tras-os-Montes e Alto Douro, Portugal; 6 Center for Research in Neuropsychology and Cognitive and Behavioral Intervention (CINEICC), Faculty of Psychology and Educational Sciences, University of Coimbra, Portugal; 7 Department of Education and Psychology, University of Aveiro, Portugal; 8 Center for Health Technology and Services Research at the Associate Laboratory RISE—Health Research Network (CINTESIS@RISE), Portugal

**Keywords:** Fear of Dependency Scale, psychometric properties, validity, reliability, older adult

## Abstract

**Background:**

Dependency is defined as a person’s inability to meet basic human needs. In the context of aging, biopsychosocial changes compromise functionality, predisposing older adults to rely on others to perform daily activities. Fear of dependency describes the fear people have of appearing and/or being weak and/or reliant on others. The Fear of Dependency Scale (FDS), developed by Adams-Price and Ralston in 2016, aims to assess the fear of dependency by measuring an individual’s attitudes toward being helped.

**Objective:**

This study presents a European Portuguese version of the FDS and examines its psychometric properties (internal consistency reliability and content validity).

**Methods:**

The translation and both cultural and linguistic adaptation of the FDS were performed by a panel of experts. A cross-sectional study was then performed to evaluate the psychometric properties (in terms of its reliability and validity) of the translated version of the FDS among a sample of 100 community-dwelling older adults.

**Results:**

The European Portuguese version of the FDS exhibited good psychometric properties. The scale’s internal consistency was 0.84.

**Conclusions:**

The European Portuguese version of the FDS is a reliable, valid, and practical instrument for assessing the fear of dependency in older adults in the Portuguese population. It can be used in the context of health care provision and research.

Population aging is a major global problem in developed societies, where 80% of older adults will live in low- and middle-income countries.^1^ From 2015 to 2050, the world’s population over 60 is expected to almost double, from 12% to 22%.^1^ In Portugal, people over 65 represent 23.8% of the population; the aging index is 182.1% (182.1 older adults per 100 young people), the longevity index (ratio of the number of people aged 75 and over to the number of people aged 65 and over) is 48.7%, and the old-age dependency ratio is 36.8%.^2-5^ These data indicate that the Portuguese population is aging and living longer.

Aging and increased longevity raise several concerns and prompt feelings of unease in older people due to the associated loss of physical and intellectual abilities, the increased susceptibility to disease, and consequently, the accumulation of deficits that can lead to dependency. Moreover, improvements in both the quality of health care and socioeconomic conditions lead to an increase in the number of chronic diseases, which tend to increase the likelihood of dependency.^6-8^

In clinical settings like hospitals and primary care, dependency is interpreted as the result of a functional incapacity, either acute or chronic, which leaves the person unable to carry out an activity that they were previously able to do on their own due to a complex combination of factors that change with age.^9,10^ As people get older, it is common to see an increase in dependency, with reported differences between age groups (65-74, 75-79, and over 80 years).^7-9^ As a multidimensional phenomenon, the factors that tend to characterize dependency are advanced age and the existence of an illness or disability with physical, psychological, intellectual, and/or sensory limitations that require the help of others to carry out activities of daily living.^6,9^ The transition to dependency entails a complex, multidimensional, and multicausal process related to an unanticipated event that arises abruptly or progressively, thereby compromising the individual’s self-care capacity and reducing one’s ability to adapt to the living context. Losses and fears tend to accompany this transition phase.^10^ Among the fears most commonly experienced by older adults are fears regarding the proximity of death, the possibility of institutionalization, and the potential for dependency.^6,11^

The fear of dependency in older adults is associated with the possibility of becoming bedridden and of undergoing changes that may range from mobility loss to loss of lucidity, loneliness, and becoming a burden to others.^6,8,12^ These kinds of fears often result from a set of life experiences and sociocultural influences rather than directly from the aging process.^6-8^ Adams-Price and Ralston^12^ defined the fear of dependency as people’s fear of appearing weak and dependent on others. This fear of appearing dependent can serve as a powerful behavioral motivator for older people, as it can result in the avoidance of being seen in public or of displaying frailty or indications of illness. In fact, the fear of appearing dependent in the eyes of others causes some older people to avoid using walking or hearing aids, which would actually increase their independence.^13^ In addition, the fear of dependency may lead some older adults to increasingly stay at home, which can lead to depression^14,15^ and the loss of functionality due to extended immobility.

The Fear of Dependency Scale (FDS) is an instrument that aims to assess how much a person fears becoming dependent in old age.^12^ To accomplish this, the FDS assesses negative attitudes toward aging, such as feeling like a burden when asking for help, preferring to stay at home rather than appearing dependent, being indebted to others, and preferring to pay someone for help rather than asking a family member. The instrument is comprised of 5 questions, which facilitates its application in both clinical practice and research. The use of the FDS is very important in comprehensive geriatric assessment as well as all contexts of care for older adults, because the fear of dependency is not only common, but also has a major impact on one’s physical and mental health.^12,16^

## Purpose

This study sought to translate and adapt the FDS into the European Portuguese language and to assess the psychometric properties of the translated scale among a sample of 100 community-dwelling older adults who live in their own homes in the North of Portugal and are not institutionalized. The study also aimed to analyze the relationship between the fear of dependency and a set of sociodemographic variables (sex, age, marital status, cohabitation), physical condition (functional independence level, functional mobility, balance, gait, risk of falling), psychological status (emotional distress [anxiety and depression]), health self-perception, and fear of falling.

## Methods

### Design

The FDS was translated, culturally adapted, and linguistically validated into European Portuguese. The scale was translated from the original language (English) into the final language (European Portuguese) by 2 bilingual translators. Cultural adaptation included the stages of translation, back-translation, review by a group of experts, and pretesting, ensuring the scale is relevant and understandable while maintaining the integrity of the items. Subsequently, the psychometric properties were examined and the associations between the fear of dependency and the following variables were explored: sex, age, marital status, cohabitation, functional independency level, fear of falling, and self-perception of health. For data collection, a protocol developed by the authors was applied, involving a questionnaire on sociodemographic and health information. All data were collected in person (interview) by the main researcher, who is a nurse, at the Older Adults’ Health Nursing Consultations in a Primary Health Care service.

### Sample

Nonprobabilistic convenience sampling was performed. The inclusion criteria for the study were as follows: having Portuguese nationality, being 65 years old or over, living in their own homes (ie, not institutionalized), and agreeing to participate voluntarily in the study by signing a consent form. Older adults with cognitive impairment were excluded. The data collection took place in January and February 2020 in health centers located in Vila Real (Northern Portugal). The study was approved by the Ethics Committee for Health of the Regional Health Administration of the North (approval no. 123/2019).

### Measures

#### Fear of Dependency Scale

The FDS was developed by Carolyn Adams-Price and Margaret Ralston in 2016 in the United States from a sample of 1424 adults aged between 45 and 99 years.^15^ In the original study that presented the development of the FDS, the authors used 5 items to measure the concept (eg, “I would rather stay home than look dependent”). Each item has 5 response options, ranging from “Strongly disagree” to “Strongly agree.” The first item (“I can ask others for help because I have helped others”) is reverse coded. The item scores are summed to calculate the total score, which ranges from 5 to 25 points, with higher scores indicating more fear of dependency.^15^

#### Functional independence

The Barthel Index is an instrument that evaluates a person’s level of independence in relation to performing 10 activities of daily living: feeding, bathing, dressing, personal hygiene, sphincter control, bladder control, using the toilet, walking, transferring from chair to bed, and going up and down stairs.^17^ The score for the original version of the Barthel Index ranges from 0 to 100 (at 5-point intervals). The minimum score of 0 corresponds to total dependency in terms of the activities of daily living, whereas the maximum score of 100 is equivalent to total independence for the same activities.^17,18^ The participants in this study were classified as functionally independent (score = 100) or functionally dependent (score <100). The Barthel Index had a high Cronbach’s alpha in this study (0.91).

#### Health self-perception

The participants’ health self-perception was assessed via a single question (“How do you think your health is compared to people of your age and sex”) with 5 response options on a Likert scale: 1 = “Very bad,” 2 = “Bad,” 3 = “Not good or bad,” 4 = “Good,” and 5 = “Very good.”

#### Falls Efficacy Scale–International

The Falls Efficacy Scale–International (FES-I) is a scale that assesses the fear of falling during the performance of daily living, physical, and social activities.^19,20^ Developed by the European Falls Prevention Network (ProFaNE), it includes 16 items rated on a 4-point Likert scale (1-4). Each item has 4 response options: “Not all concerned” (1), “Somewhat concerned” (2), “Fairly concerned” (3), and “Very concerned” (4). The item scores are added, leading to a score ranging from 16 to 64 points, with higher scores indicating more fear of falling. The FES-I had a high Cronbach’s alpha in this study (0.95).

#### Timed Up and Go test

The Timed Up and Go (TUG) test assesses balance, functional mobility, gait, and risk of falling in older adults. The TUG involves the participant getting up from a seat with 2 arm rests that is between 44 and 46 cm in height. Without any aids, the participant is then required to walk 3 m forward, make a 180° turn, and return to the chair and sit down. To initiate this test, the participant is given the instruction “go” and then timed in seconds from the moment of the instruction to resting back into the chair. Two tests were performed for each participant, and the best performance was recorded. When a participant performs the test in less than 10 seconds, it indicates independence in everyday activities, whereas a time over 30 seconds indicates changes in mobility and dependence in most everyday activities.^21,22^

#### Hospital Anxiety and Depression Scale

The original Hospital Anxiety and Depression Scale (HADS) was developed by Zigmond and Snaith^23^ to assess anxiety and depression. The scale was validated for the Portuguese population by Pais-Ribeiro et al.^24^ It comprises 14 items rated on a 4-point Likert scale ranging from 0 to 3, with 7 items assessing depression symptoms and 7 items assessing anxiety symptoms. The scores for the scale and subscales are obtained using the sum of the corresponding items. Thus, the total score (emotional distress) ranges from 0 to 42, while the depression and anxiety subscale scores range from 0 to 21. The higher the scores, the higher the levels of emotional distress, depression, and anxiety. The Cronbach’s alphas were higher than 0.70 for the overall scale (0.82), the depression subscale (0.70), and the anxiety subscale (0.73).

### Study Procedures

The process of translation, cultural adaptation, and validation for the Portuguese population followed Vilelas’ guidelines and included the following stages: Phase 1, translation of the FDS scale from the source language (English) into the final language (European Portuguese) by 2 bilingual translators; Phase 2, synthesis of the translations carried out by the initial translators by an external observer; Phase 3, 2 retroversions (back-translations) of the scale into the scale’s source language; and Phase 4, meeting of a committee of experts, made up of health professionals, language professionals and the translators, and a single final translation was created.^25^ Written reports were produced at each stage, documenting all the phases of the process that led to the pre-final version of the scale.

The translated version was pretested on the target population (ie, community-dwelling older adults). Ten older adults over the age of 65 participated (5 women and 5 men), from the Older Adults’ Health Nursing consultation at a health center. The objectives of the study were explained to the participants, guaranteeing the confidentiality and anonymity of the data. After analyzing the data, there was no need to make any changes, as the scale developed proved to be brief and very easy to understand.

For the validation of the scale, the population considered was 100 older adults. A respondent-to-item ratio of 20 was used for determining the sample size, above the minimum suggested by Boateng et al^26^ who recommend at least 10 participants for each scale item. The research was complemented with a set of sociodemographic, physical, and psychological variables since there is little research on fear of dependency, and in a study by the developers of the FDS, fear of dependency was a predictor of depression after taking health, age, and disability into account.^15^

### Data Analysis

Continuous variables were described using descriptive statistics (minimum, maximum, median, mean, and standard deviation). Frequencies (n and %) were used to describe the categorial variables. The European Portuguese FDS’s reliability was assessed via inter-item correlations, corrected item-total correlations, and Cronbach’s alpha. It was also verified whether the exclusion of an item would improve the scale’s Cronbach’s alpha. The following rules of thumb were used to confirm the reliability of the FDS: inter-item correlations higher than 0.30, corrected item-total correlations higher than 0.50, and Cronbach’s alpha higher than 0.70.^27^ To identify the variables associated with the FDS score, Student’s *t* test, analysis of variance (ANOVA), Spearman’s correlation coefficient, and the Pearson correlation coefficient were used. A linear regression model was developed to study the influence of the FDS score on a measure of anxiety and depression (HADS),^24^ adjusted for age and gender. The assumptions concerning the statistical tests were assessed and validated. A significance level of 5% was considered for the statistical tests. SPSS version 26 for Windows software (IBM Corp) was used for all the statistical analyses.

## Results

The sample comprised 100 older adults aged 65 to 94 years (mean [SD]: 77.1 [6.9]), 49% of whom were women. Most participants were married or lived in a de facto union (73%), and most were functionally independent (88%). As for perceived health status, 46% considered their health to be good or very good, 25% considered it to be bad or very bad, and 29% not good or bad (Table 1). The FES-I scores ranged from 16 to 64, with a mean (SD) of 28.2 (11.7). The times for the TUG test ranged from 5 to 50 seconds, with a mean of 12.06 (7.83) seconds. As for the HADS scores, the mean was 6.5 (SD = 3.7, range = 0-18) for depression and 6.2 (SD = 3.7, range = 0-17) for anxiety.

**Table 1.** Sociodemographic and General Assessment Data (N =100).

Variable	n (%)
Sex	
Male	51 (51)
Female	49 (49)
Age	
65-74 years	37 (37)
75-79 years	34 (34)
≥80 years	29 (29)
Marital status	
Single	2 (2)
Married/de facto union	73 (73)
Widowed	23 (23)
Divorced/separated	2 (2)
Living alone	
No	84 (84)
Yes	16 (16)
Functional independency	
Dependent	12 (12)
Independent	88 (88)
Health self-perception	
Very bad	3 (3)
Bad	22 (22)
Not good or bad	29 (29)
Good	35 (35)
Very good	11 (11)

The inter-item correlations of the FDS (Table 2) showed low correlations between item 1 and the other items, where the correlations were lower than 0.16 and not significantly different from zero (*P* > .05). By contrast, the correlations among items 2, 3, 4, and 5 were higher than 0.50 and significantly different from zero (*P* < .001).

**Table 2.** Inter-Item Correlations of the Fear of Dependency Scale (N = 100).

Item	Item 1, *r* (*P*)	Item 2, *r* (*P*)	Item 3, *r* (*P*)	Item 4, *r* (*P*)
Item 1	—			
Item 2	−0.033 (.742)	—		
Item 3	−0.004 (.966)	0.477 (<.001)	—	
Item 4	0.159 (.113)	0.634 (<.001)	0.576 (<.001)	—
Item 5	0.037 (.713)	0.530 (<.001)	0.656 (<.001)	0.551 (<.001)

Corrected item-total correlations are reported in Table 3, confirming the low correlations between item 1 and the other items. The corrected item-total correlation of item 1 (correlation between item 1 and the scale score that excludes item 1) was close to zero (0.051). Furthermore, the exclusion of item 1 increased the FDS’s Cronbach’s alpha from 0.74 to 0.84. Thus, it was decided to exclude item 1 from the scale. The analysis without item 1 showed high reliability (Cronbach’s alpha = 0.84) and high corrected item-total correlations (>0.64). The Cronbach’s alpha did not increase with the exclusion of any other items.

**Table 3.** Corrected Item-Total Correlation, Cronbach’s Alpha Without the Item, and Cronbach’s Alpha of the Fear of Dependency Scale (N = 100).

Items	With 5 items		Excluding item 1	
	Correlation*	Alpha**	Correlation*	Alpha**
1. I can ask others for help because I have helped others.	0.051	0.841	—	—
2. If I ask for help, then I am a burden to others.	0.575	0.666	0.643	0.812
3. I would rather stay home than look dependent.	0.618	0.651	0.677	0.799
4. I feel obligated when others help me.	0.707	0.608	0.698	0.789
5. I would rather pay someone to help me than ask for help.	0.640	0.636	0.686	0.795
Cronbach’s alpha		0.739		0.841

* Corrected item-total correlation.

** Cronbach’s alpha without the item.

The FDS score was calculated using the sum of items 2, 3, 4, and 5; the higher the score, the higher the fear of dependency. The sample’s FDS scores ranged from 4 to 20, with a median of 14.5 and a mean of 13.6 (SD: 4.9). Table 4 shows that the mean (SD) FDS score was significantly higher in the participants who were married or in a de facto union when compared to those who were single, widowed, or divorced/separated (14.3 [4.9] vs 11.7 [4.4], *P* = .02). The older participants (≥80 years) presented lower FDS scores (11.8 [4.3]) than the younger participants (65-74 years: 13.9 [4.6]; 75-79 years: 14.7 [5.4]), although the differences were not statistically significant (*P* = .052). No statistically significant differences were found regarding living alone (*P* = .106), sex (*P* = .371), or functional independence (*P* = .216).

**Table 4.** Relationship Between the Fear of Dependency Scale Score and Demographic Variables (N = 100).

Variable	FDS score, mean (SD)	*P*
Gender		.371^a^
Male (n = 51)	13.14 (5.12)	
Female (n = 49)	14.02 (4.69)	
Age		.052^b^
65-74 years (n = 37)	13.89 (4.62)	
75-79 years (n = 34)	14.74 (5.40)	
≥80 years (n = 29)	11.79 (4.29)	
Marital status		.020^a^
Single, widowed, divorced/separated (n = 27)	11.70 (4.44)	
Married/de facto union (n = 73)	14.26 (4.93)	
Living alone		.106^a^
No (n = 84)	13.92 (4.93)	
Yes (n = 16)	11.75 (4.52)	
Functional independence		.216^a^
Dependent (n = 12)	11.92 (4.56)	
Independent (n = 88)	13.80 (4.94)	

Abbreviation: FDS: Fear of Dependency Scale.

a *P* value of Student’s *t* test.

b *P* value of the analysis of variance.

The FDS score was negatively correlated with health self-perception (Spearman’s *r* = −0.31, *P* = .002) but positively correlated with the HADS depression score (Pearson *r* = 0.20, *P* = .051). No significant correlations were found between the FDS score and the fear of falling (FES-I; Pearson’s *r* = 0.11, *P* = .27), mobility and balance (TUG; Pearson’s *r* = −0.14, *P* = .17), HADS total score (Pearson’s *r* = 0.12, *P* = .27), or HADS anxiety score (Pearson’s *r* = 0.00, *P* = .98).

The linear regression model showed that when adjusted for sex and age, the FDS had a positive influence on the HADS depression score (β = .227, *B* = 0.171, *P* = .03). Sex (β = −.023, *B* = −0.172, *P* = .82) and age (β = .163, *B* = 0.090, *P* = .12) did not significantly influence the HADS depression score. The 3 independent variables in the regression model (sex, age, and HADS depression score) explained 6.6% (*R*^2^ = 0.066) of the variability in the HADS depression score.

## Discussion

This study sought to translate and adapt the FDS into the European Portuguese language and to assess its psychometric properties. The study also aimed to analyze the relationship between the fear of dependency and a set of sociodemographic, physical, and psychological variables. Overall, along with the scale presenting good psychometric properties, the fear of dependency was found to be higher in the married or cohabiting participants with less positive health self-perception. Higher fear of dependency was also found to be a predictor of depressive symptomatology.

The psychometric properties of the European Portuguese version of the FDS for community-dwelling older adults were analyzed in terms of reliability and validity (internal consistency reliability and content validity). The internal consistency of the scale assessed with the 5 items through Cronbach’s alpha (0.74) is nearly identical to the value of the original scale (0.72).^12^ The results showed very good internal consistency (Cronbach’s alpha = 0.84) after the elimination of item 1 (“I can ask others for help because I have helped others”) from the scale.^28^ This item was eliminated for several reasons. First, the inter-item and item-total correlations with the other items were close to zero, indicating that this item may not assess the same content as the other items.^26^ In addition, the exclusion of item 1 considerably improved the reliability of the scale (Cronbach’s alpha increased from 0.74 to 0.84). Second, item 1 does not express a person’s fear of being dependent due to having helped others in the past. Third, shorter assessment scales are always preferred in the global assessment of older adults due to their applicability.^29^ For these reasons, item 1 was excluded from the FDS and validation was performed with 4 items.

In terms of the study’s second objective, the FDS score was found to be negatively correlated with health self-perception (Spearman’s *r* = −0.31, *P* = .002) but positively correlated with the HADS depression score (Pearson’s *r* = 0.20, *P* = .051), indicating that older adults with negative health self-perception and depressive symptoms exhibit a greater fear of dependency. Prior studies have shown that older people who hold negative stereotypes of aging, such as the assumption that they are more dependent on others, tend to develop worse health and well-being than similarly aged individuals who reject such stereotypes.^30-32^ The linear regression model also showed the fear of dependency to be a predictor of depression (β = .227, *B* = 0.171, *P* = .03). A similar finding was reported by Adams-Price,^15^ who applied the FDS to 1424 participants aged 45 to 99 years in the United States. After regression analyses, the author concluded that the fear of dependency was a strong predictor of depression when considering health status, age, and disability.

In this study, the older participants (aged ≥80 years) had lower FDS scores than the younger participants, although the differences did not reach statistical significance. This result may be related to the fact that as people age, they may develop greater acceptance of the inevitable nature of aging and the possibility of facing illness and physical limitations, leading to fewer negative emotions and more optimism regarding aging and the future.^33,34^ Such acceptance may lead to a more relaxed attitude toward dependency, thereby reducing the associated fears. In addition, acceptance facilitates the development of greater resilience when it comes to dealing with both physical and emotional challenges.^35^ This resilience may help older adults face the fear of dependency in a more positive way, which accords with the findings of other studies where resilience was shown to be an important protector of mental health against the effects of aging.^36,37^ It is important to highlight, however, that the level of dependency on others constantly changes throughout life as a function of individual circumstances and environmental contexts. In Portugal, for instance, a population-based study involving very old individuals (aged 100 and over) found that community-dwelling older adults were less dependent and had better cognitive health than those living in a nursing home.^38^ Dependency tends to increase with age, although this does not mean that older adults are more dependent. Indeed, some studies have shown great heterogeneity and variety in the levels of dependency, and many older adults maintain their independence until the end of their life.^7,9^

This study revealed the absence of statistically significant differences between the fear of dependency and functional independency, sex, or fear of falling, although the FDS score was found to be significantly higher in those participants who were married or in a de facto union. As the FDS is a recently developed measure, there are few studies available to explain this relationship, although it may be related to the fact that those who are in an emotionally close relationship may not want to appear dependent, generate work, or be a burden to the people they live with.^12^ The social representation of dependency may also contribute to explaining the fear of dependency in the married or cohabiting participants, as being cared for by someone involves physical and emotional dimensions, including family and social relationships, which are driven by positive and negative feelings. When one spouse becomes dependent, the independence of the other person and the marital relationship may be affected, as the healthy person may have to take on new caregiving responsibilities and lose some of their independence and freedom. Moreover, significant financial concerns related to dependency may also explain the fear of becoming dependent.^6,8^

It must be acknowledged that this study has some limitations. First, it is based on a convenience sample from a specific geographical area, which reduces the generalizability of the results. In addition, the data collection protocol was administered by the researcher in an interview context, which may have influenced the participants to respond in socially acceptable ways. Yet despite these limitations, this study contributes to scientific knowledge concerning the fear of dependency assessment in community-dwelling older adults in both research contexts and clinical practice, as it has revealed that the European Portuguese version of the FDS has good psychometric characteristics in terms of reliability and validity. To the best of the authors’ knowledge, this scale is the only one available in Portugal to assess this construct. It is a short scale that can be readily incorporated into research protocols and routine health and social care, and the information it provides might be used to intervene in modifiable cognitive factors that influence psychological distress among older adults.

The main aim of this study was to provide a validated instrument to support nurses in clinical decision-making regarding the fear of dependency, thereby individualizing nursing interventions with a positive impact on reducing the fear of dependency and increasing the quality of life of older adults. Through being a short, reliable, valid, and easily applicable instrument to assess the fear of dependency, the European Portuguese FDS may offer added value for clinical practice given the limited time and resources typically available in different care settings. The validation of the FDS in other populations/settings and countries is recommended for future studies.
